# Effects of Hydrocodone Rescheduling on Pain Management Practices Among Older Breast Cancer Patients

**DOI:** 10.3390/curroncol32110593

**Published:** 2025-10-23

**Authors:** Chan Shen, Mohammad Ikram, Shouhao Zhou, Roger Klein, Douglas Leslie, James Douglas Thornton

**Affiliations:** 1 Department of Surgery, College of Medicine, The Pennsylvania State University, Hershey, PA 17033, USA; mikram2@pennstatehealth.psu.edu; 2 Department of Public Health Sciences, College of Medicine, The Pennsylvania State University, Hershey, PA 17033, USA; szhou1@pennstatehealth.psu.edu (S.Z.); dleslie@pennstatehealth.psu.edu (D.L.); 3 Penn State Cancer Institute, Hershey, PA 17033, USA; 4 Department of Economics, Rutgers University, New Brunswick, NJ 08901, USA; klein@economics.rutgers.edu; 5 Department of Pharmaceutical Health Outcomes and Policy, College of Pharmacy, University of Houston, Houston, TX 77204, USA; jdthornt@central.uh.edu; 6 Prescription Drug Misuse Education and Research (PREMIER) Center, College of Pharmacy, University of Houston, Houston, TX 77204, USA

**Keywords:** pain management, breast cancer, opioid and non-opioid pharmacotherapy, hydrocodone rescheduling, SEER-Medicare data

## Abstract

Hydrocodone, a commonly prescribed opioid, was rescheduled from Schedule III to Schedule II in October 2014, imposing stricter prescribing regulations. While prior studies have examined its effects in general populations, its impact on breast cancer patients remains unclear. We evaluated changes in pain management among older women with early-stage breast cancer following this policy change. Using SEER-Medicare data from 2011–2019, we identified a retrospective cohort of 52,792 women aged ≥66 years. We assessed trends in the use of hydrocodone, non-hydrocodone opioids, NSAIDs, and antidepressants before and after rescheduling. Hydrocodone use declined from 55% to 40%, while non-hydrocodone opioid use increased from 43% to 50%. Multivariable logistic regression adjusted for demographic and clinical factors confirmed a significant decrease in hydrocodone use (AOR: 0.81, 95% CI: 0.75–0.86) and an increase in non-hydrocodone opioid use (AOR: 1.25, 95% CI: 1.21–1.30). Hydrocodone dosage also declined, while non-hydrocodone opioid dosages remained stable. No significant changes were observed in NSAID or antidepressant use. These findings suggest that hydrocodone rescheduling significantly altered opioid prescribing patterns, reducing hydrocodone use and prompting a shift toward alternative opioids. Further research is warranted to evaluate the appropriateness and outcomes of such shifts in cancer pain management.

## Simple Summary

We analyzed a large national database to assess hydrocodone rescheduling’s impact on hydrocodone, non-hydrocodone opioids, and non-opioid pain management among 52,792 women aged ≥66 years with early-stage breast cancer from 2011 to 2019. Results showed a significant decrease in hydrocodone use and dosage, alongside a significant increase in the use of non-hydrocodone opioids, with no significant changes in nonsteroidal anti-inflammatory drugs (NSAIDs) or antidepressant use. These findings suggest policy-driven reductions in hydrocodone use led clinicians to shift to other opioids, with minimal impact on non-opioid strategies, warranting further research on the appropriateness and outcomes of evolving opioid prescribing patterns.

## 1. Introduction

Opioids are a potent class of analgesic drugs, that have been frequently prescribed for pain management over the last few decades [1,2]. Overuse, misuse, and long-term use of opioids are associated with severe negative consequences such as dependency, psychotic disorders, and death [1,2,3,4]. According to the Centers for Disease Control and Prevention, since 2000 there has been a 200% increase in the rate of opioid-related overdose death [5]. Among opioids, hydrocodone has been the most commonly prescribed drug in the United States for a decade [6,7]. Intending to prevent their misuse, the US Drug Enforcement Administration rescheduled hydrocodone products from Schedule III to Schedule II under the Controlled Substances Act [8]. The act was effective from October 2014. The key difference between Schedule III and II is that Schedule II requires a new prescription for each fill and cannot be prescribed over phone and facsimile transmission [8,9].

Many studies have evaluated the effect of hydrocodone rescheduling on different demographics including surgery patients, chronic hydrocodone users, prescribers, and pharmacies [9,10]. Most studies found that rescheduling was associated with a decrease in prescribing and dispensing hydrocodone [11,12,13]. For example, Tran et al. reported that hydrocodone claims decreased from 45% to 34% in new users after rescheduling [12]. Karami et al. (2023) reported that quarterly hydrocodone dispensing decreased by 177 million dosages after rescheduling [14].

Despite the findings of these studies, little is known about the effect of rescheduling on breast cancer patients. Breast cancer remains one of the most common cancers in women in the United States [15]. According to the Center for Disease Control and Prevention, the rate of new breast cancer cases remains near or over 120 per 100,000 women between 1999 and 2020 in the US [16]. When detected early such as in the localized stage, the 5-year survival rate of breast cancer can be as high as 99% [17]. Hence appropriate pain management with minimal long-term negative impacts is especially important for early-stage breast cancer patients. Effective pain management is critical in breast cancer patients. A substantive proportion of breast cancer patients experience pain and they are generally associated with multiple facets of cancer itself and its treatments such as tumor pain, surgery, radiation, chemotherapy, hormonal therapy, and mental stress [18,19]. Older breast cancer patients are one of the most vulnerable groups of patients because of their advanced age and potentially larger number of comorbidities, which poses further challenges in appropriate pain management.

Previously, a pioneer study by Gibson et al. examined the effect of rescheduling on breast cancer patients aged over 18 after surgery where the authors reported that they are less likely to receive long- and short-term prescriptions of opioids [20]. However, the study did not differentiate between the use of hydrocodone and non-hydrocodone opioids. It is plausible that rescheduling may affect the use of hydrocodone and non- hydrocodone very differently, and the insights on the differential impacts of policy on hydrocodone and non-hydrocodone can be important in designing future policies. Moreover, the effect of rescheduling on the dose of hydrocodone and other opioids was not studied. Furthermore, there is paucity of research about the impact of rescheduling on the use of alternative non-opioid pain management pharmacotherapies such as NSAIDs and antidepressants in older early-stage breast cancer patients [21].

In this study, we aim to gain a more comprehensive understanding of how the rescheduling of hydrocodone affected the pain management of older early-stage breast cancer patients, including the uptake and dosing of both hydrocodone and non-hydrocodone opioids, and also the use of alternative non-opioid pharmacotherapies.

## 2. Materials and Methods

### 2.1. Data Source

The data for this study were derived from the Surveillance, Epidemiology, and End Results (SEER) merged with Medicare claims for patients above 65 years of age. The SEER registry supported by the National Cancer Institute (NCI) is a highly regarded source for population-based cancer research, encompassing over 35% of the U.S. population [22,23]. It offers comprehensive data on patient demographics, primary tumor sites, stage at diagnosis, and survival outcomes. By linking SEER data with Medicare claims, the dataset is enriched with additional information on healthcare utilization before and after cancer diagnosis for patients covered by Medicare, including Parts A, B, and D. Since 2007, Medicare Part D claims have provided detailed records on pharmaceutical prescriptions including prescription opioids and other medications.

### 2.2. Study Cohort

The study cohort included older females (aged ≥ 66 years) diagnosed with early-stage incident breast cancer between 1 January 2011, and 31 December 2018, with follow-up extending for one year, ending in 2019. This timeframe encompasses the policy change regarding hydrocodone rescheduling in 2014 and excludes the COVID-19 pandemic period beginning in 2020. Early-stage breast cancer patients were identified as those diagnosed with AJCC 6th Edition Stage I breast cancer and treated with primary surgery. The baseline period was defined as the one year prior to cancer diagnosis, while the follow-up period was the one year post-diagnosis. Patients with autopsy reports or death certificates during these periods were excluded. Additionally, patients were required to have continuous enrollment in Medicare Parts A, B and D, without health maintenance organization (HMO) coverage, during both the baseline and follow-up periods. This ensured complete records for identifying pre-existing comorbidities, medications related to pain management, and cancer treatment during the study timeframe.

### 2.3. Utilization of Pharmacotherapy for Pain Management

We identified the use of hydrocodone and non-hydrocodone opioids by referencing their generic names. Non-hydrocodone opioids included codeine, dihydrocodeine, fentanyl, hydromorphone, buprenorphine, methadone, levorphanol, meperidine, morphine, opium, oxycodone, oxymorphone, tapentadol, and tramadol. Similarly, the use of NSAIDs and antidepressants was determined based on their generic names [24].

The doses of opioids were converted to Morphine Milligram Equivalents (MMEs) by assigning MMES for each opioid based on the conversion algorithm endorsed by CDC [25]. Average daily MME was calculated accounting for days’ supply and time periods with active opioid prescriptions.

### 2.4. Patient Characteristics

The patient demographic characteristics considered included age at diagnosis and race/ethnicity (African American, Hispanic, non-Hispanic White, Others). Medicaid dual eligibility (yes, no) was used as an indicator of socioeconomic status. The Charlson comorbidity score [26], based on healthcare utilization during the baseline period, was used to assess overall health status, with a specific indicator for depression included due to its significant impact on pain perception and management. Additionally, we accounted for the cancer treatments received (radiation, chemotherapy, immunotherapy, and hormonal therapy) as these treatments can substantially influence pain management strategies.

### 2.5. Statistical Analysis

Descriptive statistics, including frequencies and percentages, were generated to summarize the characteristics of the patient population. Monthly time trends in the percentage of patients using hydrocodone, non-hydrocodone opioids, NSAIDs, and antidepressants during the 12 months following diagnosis from 2011 to 2019 were illustrated in graphical figures. Additionally, we depicted the monthly trends in average daily MME for hydrocodone and non-hydrocodone opioids over the same period.

To evaluate factors associated with hydrocodone use, we employed multivariable logistic regression models. These models incorporated a binary indicator to distinguish periods before and after the rescheduling of hydrocodone in October 2014, along with a continuous variable representing the time trend in months, allowing for the assessment of both policy impact and overall temporal trends in hydrocodone use. Additionally, we controlled for patient characteristics, including age, race, Medicaid dual eligibility, depression, Charlson comorbidity index, and cancer treatments in the logistic regression models. Similar multivariable logistic regression models were applied to assess the use of non-hydrocodone opioids, NSAIDs, and antidepressants. We reported the adjusted odds ratios (AORs) with 95% confidence intervals (CIs) and *p*-values for all model parameters.

For the analysis of hydrocodone and non-hydrocodone MMEs, we utilized ordinary least squares regression. This analysis included adjustments for patient characteristics, the pre- vs. post-policy indicator, and the continuous time trend. Parameter estimates, standard errors (SEs), and *p*-values were provided for these models.

As a sensitivity analysis, we applied segmented time-series logistic regression models incorporating terms for the pre-policy trend, a level-change indicator at the time of policy implementation, and an interaction term capturing changes in slope after the policy. This framework enabled estimation of both the immediate effect of the 2014 rescheduling and differences in the rate of change in pain medication use before versus after the policy.

Statistical analyses were conducted using SAS version 9.4 (SAS Institute, Cary, NC, USA). All statistical tests were two-sided. This retrospective observational study was approved by the Institutional Review Board.

## 3. Results

A total of 52,792 patients with localized stage breast cancer were identified, of whom 24,052 were hydrocodone users. The majority of these patients were White (83.0%), had received radiation therapy (57.7%), and underwent hormonal treatment (89.3%). A higher percentage of patients in the hydrocodone group received cancer treatments, including radiation, chemotherapy, immunotherapy, and hormonal therapy. The detailed sample descriptives are provided in Table 1.

**Figure 1:**
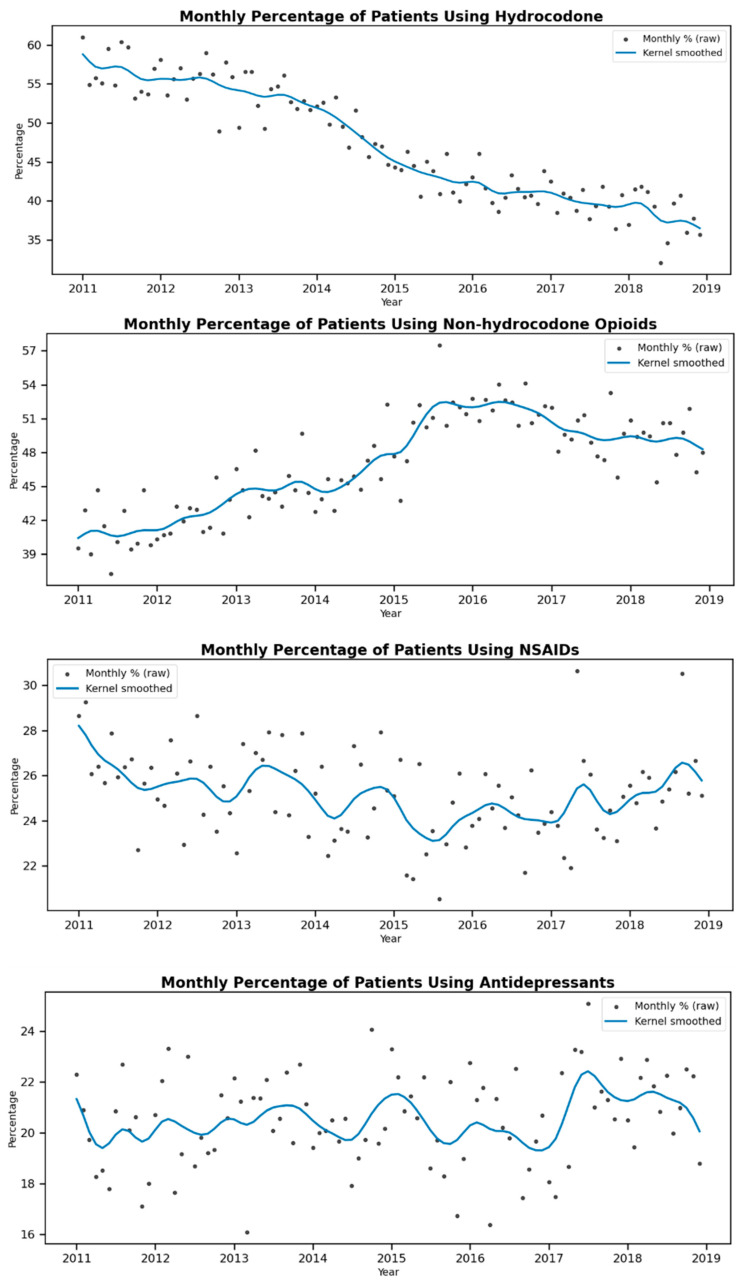
Monthly percentage of patients using hydrocodone, non-hydrocodone opioids, NSAIDs, and antidepressants with kernel smoothed trend over the raw monthly data points.

Figure 1 illustrates a substantial decrease in the percentage of patients using hydrocodone following the rescheduling of hydrocodone in late 2014. Concurrently, there was a noticeable increase in the percentage of patients using non-hydrocodone opioids during the same period. The percentages of patients using NSAIDs and antidepressants remained relatively stable throughout the policy change.

**Figure 2:**
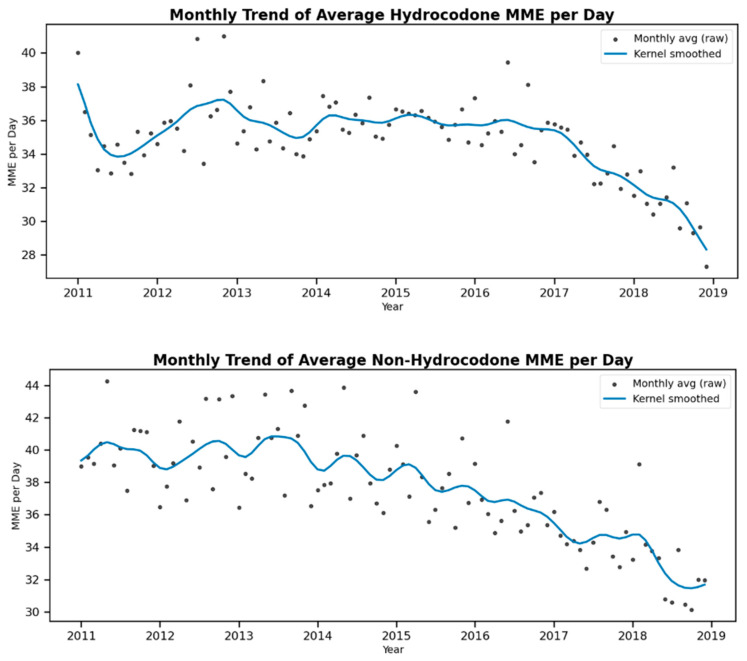
Monthly trends of opioid MMES per day for hydrocodone and non-hydrocodone opioids with kernel smoothed trend over the raw monthly data points.

Figure 2 illustrates that the daily MME of hydrocodone noticeably decreased after 2016, while the daily MME of non-hydrocodone opioids demonstrated a more gradual decrease throughout the study period.

Multivariable logistic regression analysis (Table 2) of hydrocodone use (yes/no) revealed a significant decrease in hydrocodone use following the policy change [AOR: 0.81, 95% CI: 0.75–0.86, *p* < 0.001], alongside an overall decreasing temporal trend [AOR: 0.91, 95% CI: 0.90–0.92, *p* < 0.001]. In contrast, the logistic regression analysis for non-hydrocodone opioid use demonstrated a significant increase following the rescheduling of hydrocodone, with an AOR of 1.25 [95% CI: 1.21–1.30, *p* < 0.001]. Additionally, the analyses showed no significant change in the use of NSAIDs (*p* = 0.139) or antidepressants (*p* = 0.659) before and after the policy change, nor were there significant overall temporal trends in the use of non-hydrocodone opioids, NSAIDs, or antidepressants, with all *p*-values above 0.35.

The multivariable regression analysis (Table 3) for hydrocodone dosage showed a significant decrease in the daily MME of hydrocodone by −1.637 after the policy change compared to prior [SE = 0.535, *p* = 0.002], alongside an overall decreasing temporal trend of −0.95 per year over the time period studied [SE: 0.117, *p* < 0.001]. We also observed a significant overall decreasing trend in non-hydrocodone daily MMEs of −1.624 per year [SE: 0.199, *p* < 0.001]; however, there were no significant changes before and after the hydrocodone rescheduling [*p* = 0.346].

Results from the sensitivity analysis were consistent with our main findings. Hydrocodone prescribing was already decreasing prior to rescheduling, and while the policy was associated with a marked immediate decline in use, the rate of decrease thereafter remained largely unchanged. In contrast, non-hydrocodone opioid use showed a gradual increase before the policy, a sharp but transient rise around the time of implementation, followed by stabilization in subsequent years, suggesting a temporary substitution effect. Trends for NSAID and antidepressant use were comparatively modest and less distinct. Full results are presented in Supplementary Materials Table S1. To further illustrate the counterfactual trend in the absence of the 2014 policy change, we used model-based predictions to estimate monthly opioid use assuming no rescheduling had occurred. As shown in Supplementary Materials Figure S1a, the fitted trajectory of hydrocodone use demonstrated a sharp decline around October 2014, followed by a continued gradual decrease, whereas the predicted no-policy scenario indicated a smoother, slower decline over time. In contrast, Supplementary Materials Figure S1b illustrates that non-hydrocodone opioid use exhibited a sharp increase around the time of policy implementation, after which the rate of increase tapered and eventually plateaued. These fitted trends visually illustrate that the 2014 rescheduling was associated with an abrupt shift in prescribing patterns, followed by stabilization over the subsequent years.

## 4. Discussion

In this study, we assessed the impact of the rescheduling of hydrocodone from a Schedule III- to a Schedule II-controlled substance in older patients with early-stage breast cancer. Specifically, we analyzed the use of hydrocodone, non-hydrocodone opioids, NSAIDs, and antidepressants before and after the rescheduling. Additionally, we examined the effects on opioid dosage by evaluating trends in daily MMEs. To our knowledge, this is the first study to report these findings in this vulnerable patient group.

Our findings demonstrated a notable shift in opioid prescribing patterns following the 2014 policy change. Hydrocodone use declined from approximately 55% to nearly 40%, while the use of non-hydrocodone opioids increased from around 43% to 50% during the same period. Multivariable logistic regression analyses confirmed that the odds of hydrocodone use significantly decreased post-policy change, whereas the odds of non-hydrocodone opioid use significantly increased. These results indicate that the rescheduling of hydrocodone had a substantial impact on pain management practices in cancer care, prompting a reduction in hydrocodone prescriptions and an increase in the use of alternative opioid medications. Among the alternative opioids, we found that tramadol, codeine, and oxycodone were the most commonly prescribed, with tramadol showing the largest increase—more than 5 percentage points—in its share of prescriptions following the significant drop in hydrocodone use after the policy change. Specifically, before the policy change, of the 16,285 patients who received any opioids, 3315 (20.3%) received tramadol, 4493 (27.5%) received oxycodone, 1945 (11.9%) received codeine, and 10,881 (66.8%) received hydrocodone. After the policy change, of the 25,057 patients who received opioids, 6439 (25.6%) received tramadol, 7814 (31.1%) received oxycodone, 3757 (14.9%) received codeine, and 13,086 (52.2%) received hydrocodone. It is important to note that during our study window, oxycodone products were Schedule II; tramadol became a Schedule IV controlled substance on August 18, 2014; and codeine varies by formulation: Schedule II when alone, Schedule III when combined at ≤90 mg per dosage unit (e.g., acetaminophen/codeine), and Schedule V when present at ≤200 mg per 100 mL in cough preparations [8]. The observed increase in use of non-hydrocodone opioids, particularly tramadol and, to a lesser extent, codeine, likely reflects substitution driven by their less restrictive scheduling, while the relatively modest increase in oxycodone prescribing is less likely a result of substitution effects. These patterns suggest that, following rescheduling, clinicians may have substituted non-hydrocodone opioids such as tramadol for hydrocodone to avoid additional administrative burden while continuing to manage pain and potentially maintain quality of life in this patient population.

Although no previous studies specifically evaluated the effects of hydrocodone rescheduling on elderly early-stage breast cancer patients, our findings align with the broader literature on other populations regarding the overall trends in hydrocodone and opioid use. A recent systematic review by Usmani et al. evaluated the impact of hydrocodone rescheduling on opioid use outcomes and found mixed evidence [27], though the majority of studies supported the conclusion that the policy led to decreased hydrocodone prescribing and increased non-hydrocodone opioid prescribing. Specifically, the review identified 24 studies reporting a decline in hydrocodone prescribing, while only 3 studies reported an increase. Additionally, 14 studies found an increase in the prescribing of other non-hydrocodone opioids, such as oxycodone, tramadol, and codeine, whereas only 4 studies reported decreases in non-hydrocodone opioid use. Thus, our findings suggest that healthcare providers managing cancer patients have responded to hydrocodone rescheduling in a manner similar to providers treating other patient populations.

It is notable that our multivariable logistic regression revealed a significant overall decreasing trend in hydrocodone prescribing, while the prescribing of non-hydrocodone opioids remained relatively stable over time. This suggests that hydrocodone rescheduling not only had an immediate effect, causing a sharp reduction in hydrocodone prescribing around the time of the policy change, but may also have heightened awareness of the potential risks associated with hydrocodone use. This increased awareness likely began during the discussions leading up to the policy change and persisted following its implementation, contributing to a sustained, gradual decline in hydrocodone prescribing throughout the study period from 2011 to 2019.

Meanwhile, although hydrocodone rescheduling led to an immediate increase in the use of non-hydrocodone opioids—likely as a substitute for hydrocodone in pain management—the overall trend in non-hydrocodone opioid use remained stable among early-stage breast cancer patients. Previous studies examining prescription opioid use in cancer patients have reported relatively stable or moderately declining trends in opioid use [28,29,30]. However, none of these studies distinguished the differential trends of hydrocodone and non-hydrocodone opioids in the context of hydrocodone rescheduling. Our findings underscore the importance of differentiating the effects of policy changes on various types of prescription opioids.

We found that the use of NSAIDs and antidepressants remained remarkably stable throughout the study period, with no significant impact from the policy change and no discernible overall time trend. There has been some speculation that the increased barriers to hydrocodone prescribing, along with heightened awareness of the potential negative effects of opioids, might lead physicians to prescribe more non-opioid pain management drugs as alternatives. However, there is very limited literature presenting mixed results [31,32].

Our findings indicate that there was no significant substitution of opioids with NSAIDs or antidepressants as alternative pain management strategies in this population. This is noteworthy, given growing evidence supporting the use of non-opioid pain management strategies, such as gabapentin, pregabalin, duloxetine, and NSAIDs, to reduce the need for opioid medications in cancer patients. For instance, a systematic review reported that gabapentin (in 8 studies) and pregabalin (in 4 studies) were associated with reduced opioid consumption after breast cancer surgery [33]. Thus, our findings suggest a potential missed opportunity to incorporate NSAIDs and antidepressants as integral components of pain management in breast cancer patients.

Our study demonstrated that the rescheduling of hydrocodone was associated with a significant decrease in the average hydrocodone daily MME, indicating that the policy not only influenced opioid uptake but also reduced dosage levels. This finding aligns with several studies in the literature, although those studies focused on different populations, such as chronic hydrocodone users, Medicare beneficiaries, and general state populations [32,34,35,36,37]. However, there are also studies, particularly among Medicaid patients and post-surgical populations, that reported either no change or an increase in hydrocodone dosage following rescheduling [12,38,39]. These mixed findings underscore the variability in outcomes depending on the study population examined.

Interestingly, our study found no significant association between hydrocodone rescheduling and the dosage of non-hydrocodone opioids, suggesting that the impact of rescheduling on hydrocodone dosage did not extend to the dosage patterns of non-hydrocodone opioids. However, our overall analysis revealed an overall decreasing trend in MMEs for both hydrocodone and non-hydrocodone opioids throughout the study period, suggesting that physicians adopted a more conservative approach to opioid prescribing, in general, over the study period regardless of the hydrocodone rescheduling. This aligns with findings from other studies that report reduced opioid dosages in various populations such as trauma and acute care patients for example [40,41,42]. Beyond the hydrocodone policy itself, U.S. opioid prescribing was already trending downward with rates leveled after 2010 and declined substantially after 2012, with further decreases following the 2016 CDC Guideline, which likely contributed to the sustained reductions in average MME we reported [43].

Our study possesses several notable strengths. First, we utilized a comprehensive cancer registry renowned for its high-quality data. Additionally, by linking cancer registry data with Medicare claims, we were able to control for cancer-related factors and capture care across diverse healthcare settings. However, our study also has some limitations. We did not have access to information on the severity of comorbidities, such as symptoms, pain levels, or mobility, which may have influenced the pain management choices. Mental health conditions, such as depression, are often under-identified in SEER-Medicare data because claims databases do not capture undiagnosed cases. Information on over-the-counter NSAIDs is not available in the SEER-Medicare claims data, therefore our results on the use of NSAIDs are limited to prescription NSAIDs. Our study also did not capture gabapentinoids or muscle relaxants, which may be used as substitutes or adjuncts to opioids. However, these agents have only modest efficacy for select pain syndromes and are not recommended as general alternatives to opioids, with gabapentinoids primarily for neuropathic pain and fibromyalgia, and muscle relaxants mainly for short-term management of acute musculoskeletal conditions [25,44,45,46,47,48]. While we controlled for a broad range of covariates, unmeasured factors, such as opioid addiction and family history, may still impact opioid use. Furthermore, our study sample was limited to individuals aged 65 years and older, potentially limiting the generalizability of our findings to younger populations. Another limitation is the lack of detailed data on patient outcomes and pain perceptions, such as pain scores. SEER–Medicare claims lack validated pain intensity and functional status measures; thus, our study cannot determine the adequacy of symptom control or functional recovery associated with changes in prescribing. As a result, we cannot definitively assess whether the observed reduction in hydrocodone prescriptions, the increase in non-hydrocodone opioid use, or the reduction in hydrocodone dosage following the policy change affected the appropriateness of pain management or the quality of life of the patients. Future research may focus on evaluating the appropriateness of pain management strategies in breast cancer patients by incorporating actual outcomes such as pain scores and quality of life measures. Despite its limitations, this study contributes to the growing body of literature on the consequences of opioid policy changes by offering critical insights into the significant impact of hydrocodone rescheduling on pain management patterns among older breast cancer patients. Our findings highlight the complexity of opioid use dynamics, including the reduction in hydrocodone use and the corresponding increase in alternative opioid products. These results underscore the need for further research to explore the broader policy implications for pain management in breast cancer patients, particularly regarding the appropriateness and long-term outcomes of shifting opioid prescribing patterns.

## 5. Conclusions

This study provides novel insights into the impact of the 2014 hydrocodone rescheduling on opioid prescribing patterns among older adults with early-stage breast cancer. Following the policy change, hydrocodone use and dosage declined significantly, accompanied by a compensatory increase in prescriptions for alternative opioids, particularly tramadol, oxycodone, and codeine. These findings suggest that clinicians adapted their prescribing practices in response to regulatory changes, prioritizing administrative feasibility while continuing to address patients’ pain management needs. However, the stable use of NSAIDs and antidepressants indicates limited uptake of non-opioid alternatives during the same period. Our results underscore the importance of differentiating among opioid types when evaluating the impact of policy interventions and highlight a potential underutilization of multimodal pain management strategies in this population. Future research should investigate the appropriateness of evolving opioid prescribing practices and their implications for patient-reported outcomes, including pain control and quality of life.

## Figures and Tables

**Table 1:** Sample description of the study.

	Total (N = 52,272)
**Age**	
66–69	12,716 (24.3%)
70–74	15,269 (29.2%)
75–79	11,225 (21.5%)
>=80	13,062 (25.0%)
**Race/Ethnicity**	
White	43,430 (83.1%)
AA	3187 (6.1%)
Hispanic	2978 (5.7%)
Other race	2677 (5.1%)
**Hydrocodone**	
Yes	23,967 (45.9%)
No	28,305 (54.1%)
**Non-Hydrocodone opioids**	
Yes	24,880 (47.6%)
No	27,392 (52.4%)
**NSAIDs**	
Yes	13,095 (25.1%)
No	39,177 (74.9%)
**Antidepressants**	
Yes	10,754 (20.6%)
No	41,518 (79.4%)
**Radiation therapy**	
Yes	30,439 (58.2%)
No	21,833 (41.8%)
**Chemotherapy**	
Yes	14,895 (28.5%)
No	37,377 (71.5%)
**Immunotherapy**	
Yes	7350 (14.1%)
No	44,922 (85.9%)
**Hormonal therapy**	
Yes	46,853 (89.6%)
No	5419 (10.4%)
**Dual Eligibility**	
Yes	9391 (18.0%)
No	42,881 (82.0%)
**Depression**	
Yes	11,196 (21.4%)
No	41,076 (78.6%)
**Charlson comorbidity**	
No	20,245 (42.3%)
1	13,422 (28.1%)
2	8104 (16.9%)
3 or more	6051 (12.7%)

**Table 2:** Multivariable logistic regression results for hydrocodone, non-hydrocodone opioids, NSAIDs, and antidepressants.

Variables	AOR	95% CI	*p*-Value
Hydrocodone Use			
Policy Change			
Post policy change	0.81	[0.75, 0.86]	<0.001
Before policy change (reference)			
Time Trend			
in 12 months	0.91	[0.90, 0.92]	<0.001
Non-hydrocodone Opioids Use			
Policy Change			
Post policy change	1.25	[1.17, 1.34]	<0.001
Before policy change (reference)			
Time Trend			
in 12 months	1	[0.99, 1.02]	0.614
NSAIDs Use			
Policy Change			
Post policy change	0.94	[0.87, 1.02]	0.139
Before policy change (reference)			
Time Trend			
in 12 months	1.01	[0.99, 1.03]	0.359
Antidepressants Use			
Policy Change			
Post policy change	1.02	[0.93, 1.12]	0.659
Before policy change (reference)			
Time Trend			
in 12 months	0.99	[0.97, 1.01]	0.604

**Table 3:** Multivariable ordinary least squares regression results for hydrocodone MMEs and non-hydrocodone opioid MMEs.

Variables		Estimate	Standard Error	*p*-Value
Hydrocodone Use				
Policy Change				
Post policy change		−1.637	0.535	0.002
Pre policy change (reference)				
Time trend				
in 12 months		−0.95	0.117	<0.001
Non-hydrocodone Opioids Use				
Policy Change				
Post policy change		−0.856	0.909	0.346
Before policy change (reference)				
Time trend				
in 12 months		−1.624	0.199	<0.001

## Data Availability

The findings of this study are based on data from the linked Surveillance, Epidemiology, and End Results (SEER)–Medicare database. This data is not publicly available. Researchers can request access to the SEER–Medicare data through the National Cancer Institute (NCI) by entering into a data use agreement. Applications for access can be submitted at https://healthcaredelivery.cancer.gov/seermedicare/ (accessed on 3 October 2025).

## Supplementary Materials

The following supporting information can be downloaded at: https://www.mdpi.com/article/10.3390/curroncol32110593/s1, Supplementary Table S1: Segmented time series logistic regression results for hydrocodone, non-hydrocodone opioids, NSAIDs, and antidepressants use. Supplementary Figure S1: Fitted trajectory of hydrocodone use and non-hydrocodone opioids use by months.
