# Supplementary material for: Effects of Hydrocodone Rescheduling on Pain Management Practices Among Older Breast Cancer Patients
Source: Curr Oncol. 2025 Oct 23;32(11):593. doi: 10.3390/curroncol32110593 (PMC12651165; doi:10.3390/curroncol32110593)
Supplement: Supplementary file 1 [file curroncol-32-00593-s001.zip › curroncol-3869964-supplementary.pdf]

Supplementary Table S1: Segmented time series logistic regression results for hydrocodone, non-hydrocodone opioids, NSAIDs, and antidepressants use.

	AOR	95% CI	p-value
**Hydrocodone**			
Time trend before policy change (per year)	0.90	[0.88, 0.92]	<0.001
Immediate Policy Change	0.82	[0.76, 0.88]	<0.001
Post-policy slope change (per year)	1.02	[0.99, 1.05]	0.30
**Non-Hydrocodone**			
Time trend before policy (per year)	1.06	[1.03, 1.09]	<0.001
Immediate Policy Change	1.21	[1.13, 1.30]	<0.001
Post-policy slope change (per year)	0.92	[0.89, 0.95]	<0.001
**NSAIDs**			
Time trend before policy change (per year)	0.98	[0.95, 1.01]	0.21
Immediate Policy Change	0.95	[0.88, 1.03]	0.25
Post-policy slope change (per year)	1.04	[1.00, 1.08]	0.03
**Antidepressants**			
Time trend before policy change (per year)	0.98	[0.95, 1.02]	0.29
Immediate Policy Change	1.03	[0.93, 1.13]	0.60
Post-policy slope change (per year)	1.02	[0.98, 1.07]	0.31

Note: "Immediate policy change" is the level change at the October 2014 hydrocodone rescheduling. "Time trend before policy change (per year)" equals 12× the monthly pre-policy slope. "Post-policy slope change (per year)" equals 12× the change in slope after the policy. Models include month-of-year fixed effects and adjust for age group, race/ethnicity, dual eligibility, depression, Charlson comorbidity, and cancer treatments.

Supplementary Figure S1: Fitted trajectory of hydrocodone use and non-hydrocodone opioids use by months.

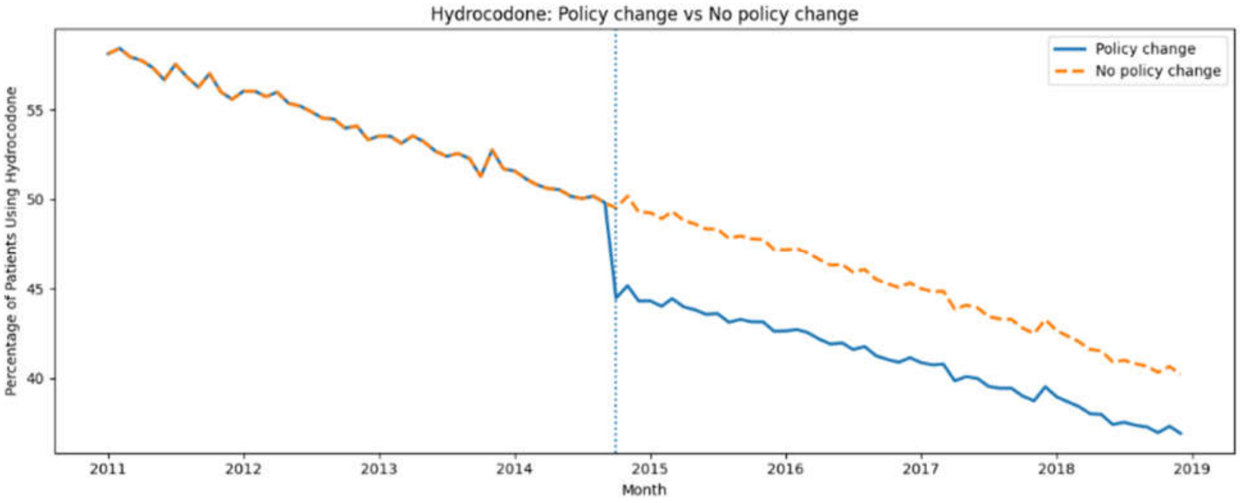

Figure S1a: Solid blue = Policy change; dashed orange = No policy change counterfactual. Dotted vertical line marks October 2014 (rescheduling). Curves are case-mix–standardized monthly predictions from patient-level logistic models adjusting for demographics, comorbidity, and cancer treatments.

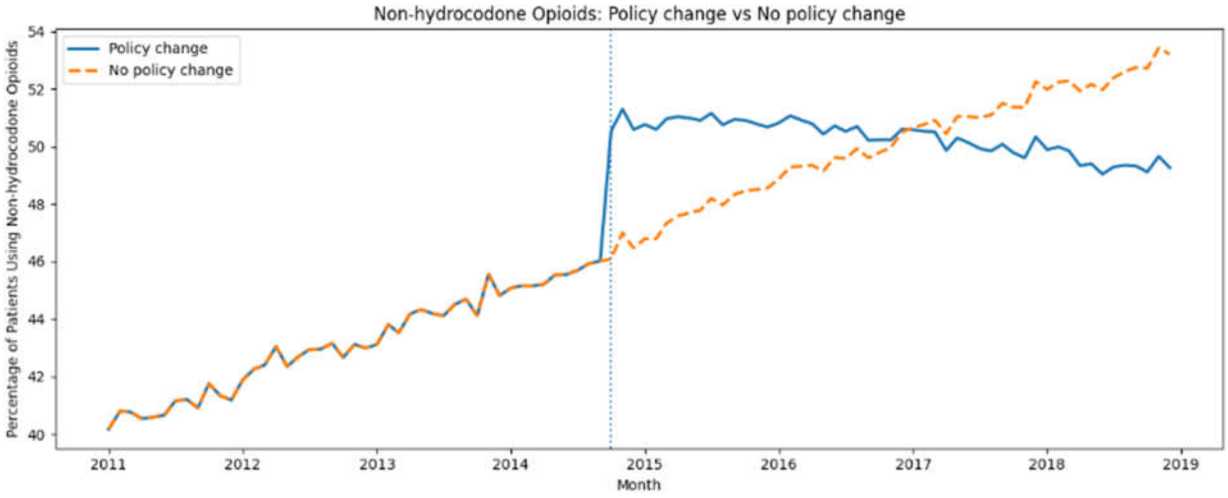

Figure S1b: Solid blue = Policy change; dashed orange = No policy change counterfactual. Dotted vertical line marks October 2014 (rescheduling). Curves are case-mix–standardized monthly predictions from patient-level logistic models adjusting for demographics, comorbidity, and cancer treatments.
